# Alternative Splicing Events Is Not a Key Event for Gene Expression Regulation in Uremia

**DOI:** 10.1371/journal.pone.0082702

**Published:** 2013-12-16

**Authors:** Marion Sallée, Michel Fontès, Laurence Louis, Claire Cérini, Philippe Brunet, Stéphane Burtey

**Affiliations:** 1 Aix-Marseille Université, INSERM UMR_S 1076, UFR Pharmacie, Marseille, France; 2 Centre de Néphrologie et Transplantation Rénale, Assistance Publique-Hôpitaux de Marseille, Hôpital de La Conception, Marseille, France; 3 Aix-Marseille Université, INSERM UMR_S 1062 UFR Médecine, Marseille, France; 4 Aix-Marseille Université, Plate-forme génomique et transcriptomique, UMR_S 910, UFR médecine, Marseille, France; Rouen University Hospital, France

## Abstract

**Background:**

The control of gene expression in the course of chronic kidney disease (CKD) is not well addressed. Alternative splicing is a common way to increase complexity of proteins. More than 90% of human transcripts are alternatively spliced. We hypothesised that CKD can induce modification of the alternative splicing machinery.

**Methods:**

During mutation screening in autosomal dominant polycystic kidney disease, we identified in mononuclear cells (PBMC), an alternative splicing event on the exon 30 of *PKD1* gene, the gene implicated in this disease. This alternative splice variant was not correlated with the cystic disease but with CKD. To confirm the association between this variant and CKD, a monocentric clinical study was performed with 3 different groups according to their kidney function (CKD5D, CKD3-5 and normal kidney function). An exon microarray approach was used to highlight splicing events in whole human genome in a normal cell model (fibroblasts) incubated with uremic serum. Alternative splicing variants identified were confirmed by RT-PCR.

**Results:**

The splicing variant of the exon 30 of *PKD1* was more frequent in PBMCs from patients with CKD compared to control. With the microarray approach, despite the analysis of more than 230 000 probes, we identified 36 genes with an abnormal splicing index evocating splicing event in fibroblasts exposed to uremic serum. Only one abnormal splicing event in one gene, *ADH1B*, was confirmed by RT-PCR.

**Conclusion:**

We observed two alternative spliced genes in two different cell types associated with CKD. Alternative splicing could play a role in the control of gene expression during CKD but it does not seem to be a major mechanism.

## Introduction

Chronic kidney disease (CKD) is associated with a dramatic increase in cardiovascular mortality [1], [2]. The high rate of mortality cannot be explained merely by the traditional cardiovascular risk factors [3]. Genomic and genetic events play a role in the physiopathology of CKD complications [4], [5]. Gene expression is controlled at two levels: transcriptional and post-transcriptional. Abnormal transcriptional activity has been reported in CKD and was related to modification in gene expression [6], modification in DNA methylation [7] or histone acetylation/deacetylation [8]. Post transcriptional modification of mRNA related to CKD has already been described as RNA silencing by micro RNA [9] or modifications of mRNA degradation [10], [11]. Alternative splicing (AS) is not mentioned in the armamentarium of gene expression regulation during CKD. Nevertheless, more than 90% of human transcripts are alternatively spliced. Some splicing events are find only in particular tissues or during specific developmental stages [12]. The splicing machinery occurs during the transcription and the spliceosome is physically linked to the transcriptional machinery [13]. The mechanisms involved in the transcriptional control reported in CKD, such as histone modifications [14] or microRNA [15] could interact with the splicing process. We hypothesize that expression of alternative spliced variants were associated with CKD and could play a part in the physiopathology of CKD.

The aim of this study was to identify a deregulation of the AS machinery during CKD.

## Subject and Methods

### Preliminary work

We have previously developed a *PKD1* mutation screening strategy based on RT-PCR amplification of the transcript using nine set of primers specific to the *PKD1* sequences [16] conducted in patients with autosomal dominant polycystic kidney disease (ADPKD). This strategy has allowed us to detect an alternatively splicing transcript of exon 30 that had not previously been identified and seems to be associated with CKD. To support this hypothesis, we screened this AS variant in twelve hemodialysed patients with autosomal dominant polycystic kidney disease (ADPKD) and eleven patients without CKD and ADPKD. This set of patients allowed us to calculate the sample size of subsequent clinical study (see statistical analysis). In addition, to exclude the relation between the AS and ADPKD we included four hemodialysed patients with ADPKD and their relatives with ADPKD but with a GFR>60 ml/mn/1.73 m^2^.

### Patients

Patients were recruited from a monocentric adult renal unit in France (Hôpital de la Conception, Marseille). Three groups were made. The first group (CKD5D group) included unrelated HD patients. The inclusion criteria were patients aged between 50 and 75 years old, thrice-weekly HD, with no intercurrent event since 3 months, dialysis treatment begun for more than two years. The second group (CKD3-5 group) included patients with chronic kidney diseases but who did not yet need HD. The inclusion criteria were patients aged between 50 and 75 years old, estimated glomerular filtration rate (eGFR) below 60 ml per minutes per 1.73 m^2^ of body surface area. The third group (control group) included healthy controls without CKD. The inclusion criteria were patients aged between 50 and 75 years old, eGFR above 60 ml per minutes per 1.73 m^2^. Acquired cystic kidney disease (ACKD) was defined by more than three cysts on both kidney dysplyed by ultrasound or more than five cysts by computed tomography. Patients with ADPKD were excluded in all groups to avoid with ACKD. The protocol for this study was approved by the local ethic committee (Comité consultatif de protection des personnes Sud-Méditerranée II), and by the scientific board (Centre d′Investigation Clinique, Marseille). This study was in compliance with the Helsinki Declaration. All the patients provided a written inform consent. The consent procedure was approved by the ethic committee.

### PBMC Isolation

Blood samples (9 ml on EDTA tubes) were obtained from each patient. In HD patients, the samples were performed before the onset of the mid dialysis session of the week. PBMCs (Peripheral Blood Mononuclear Cells) were isolated by density gradient centrifugation with Ficoll-plaque solution (GE Healthcare, Uppsala, Sweden).

### RNA extraction, reverse transcription and PCR

Total RNA was extracted from PBMC using TRIZOL reagent (Invitrogen) according to the manufacturer's instruction. Reverse transcription using random primers and oligo dT was performed on 1 µg of total RNA of each sample using the superscript II transcriptase™ (Invitrogen) for cDNA synthesis. The RT-PCR reactions were performed as described in Burtey et al [16]. A nested PCR strategy was used in order to assess the specificity of the splicing variant. We designed two oligonucleotides [AB21F: 5′-CAGCTCCGCCAACTCCGCCAACT (nt 8875 of L33234 in GenBank), AB36B: 5′-GGACAGGAGCCACGCCAACACTCAC (nt 10981)] which specifically amplify a region of the *PKD 1* transcript encompassing exons 23–35. This primer pair was used for the first-round amplifications. Products of this first-round amplification were then diluted by 1/10 000, and the internal region encompassing exons 29–31 was amplified using the following primer pair: [SAB21F6: 5′-CTGCCTCTTCCTGGGCGCC (nt 10075), SAB21B6: 5′- GCGTGGAGGCCTGAGAACG (nt 10374)]. PCR reactions were carried out using 35 cycles. PCR products were deposited at the top of a 1.5% agarose gel stained with ethidium bromide and subjected to electrophoresis. Amplicons were visualised by UV.

### Fibroblast cell culture

Human fibroblast cells were obtained from human skin after collagenase digestion as previously described [17]. The primary fibroblast cell line was produced from a female patient after she gave her informed consent for taking the cells and derived a cell line for scientific purpose. We did not ask an ethical review from our local board. 10^5^ cells were seeded on 25 cm^2^ cell culture flasks (BD falcon) and grown in DMEM medium with 10% of serum and 1% of penicillin streptomycine under standard cell culture conditions (humidified atmosphere, 5% CO2, 37°C). Cells were detached when they were confluent with a 0.05% trypsin solution and subcultured until the ten passage maximum. Fibroblasts were subcultured in two different media for 96 hours. Media were changed every 48 hours. Each media used was composed by 10% of serum, 1% of penicillin streptomycine and 89% of DMEM. Uremic serum was obtained by pooling sera of HD patients. Sera were collected from remainder plasma from HD patients before the mid dialysis session. Pooling sera was used to limited the effect of each serum. Three different pools (named 1, 2, 3) were made to obtained independent experiments. Each pooled sera consisted in a mix of at least 40 different sera each. These pooled sera were decomplementized (56°C for 45 minutes) and filtrated (0.2 µm). Fibroblasts were subcultured in either uremic serum 1, 2 or 3. Human normal serum was obtained from Lonza®. This serum was decomplementized and filtrated in the same condition than uremics were.

### DNA microarray analysis

Six RNA samples were obtained from cultured fibroblast using trizol reagent as described above. Two conditions were tested: uremic sera and normal sera. Three biological replicates were obtained for each condition. For uremic condition, RNAs were obtained from cell cultured in the three different pools of sera (1, 2 and 3). For normal condition, RNAs were obtained from cell cultured in human normal serum. RNA concentration was determined using a NanoDrop® Spectrophotometer (Thermoscientific, Wilmington, DE), and RNA integrity was tested with capillary gel-electrophoresis using RNA 6000 Nano Chips (Agilent Technologies, Santa Clara, CA), analyzed on the Agilent 2100 BioAnalyzer®. RNA was stored at −80 °C until further use. Total RNA was amplified and labeled using the Quick Amp Labeling (one color) kit® (Agilent Technologies, Santa Clara, CA). The hybridization on microarray was done using 2×400 K Agilent Human SurePrint G3 exon microarray slides (Agilent G4848A) according to the manufacturer's instructions. The arrays were scanned on an Agilent DNA microarray scanner and processed using the Feature Extraction Software® 10.7.3.1 (Agilent Technologies, Santa Clara, CA). The use of this software involved automatic grid positioning, intensity extraction (signal and background) and quality control. Data were treated with GeneSpring® 11.5.1 (Agilent Technologies, Santa Clara, CA). After quartile-based normalization, unsupervised analyses were done by hierarchical clustering using data median-centered on probe sets; Pearson uncentered correlation, and centroid linkage clustering. To identify AS variants significantly influenced by the uremic condition versus normal condition, supervised analyses were done by Mann-Whitney test between normal and uremic conditions. All statistical tests were done without any correction at the 5% level of significance to increase the number of AS highlighted at the cost of loosing sensibility. The data discussed in this publication have been deposited in NCBI's Gene Expression Omnibus and are accessible through GEO Series accession number GSE 41030 (http://www.ncbi.nlm.nih.gov/geo/query/acc.cgi?acc=GSE41030).

### Confirmation of AS variant by RT-PCR

Oligonucleotides selection was made with NCBI/primer blast (http://www.ncbi.nlm.nih.gov/tools/primer-blast). PCR primers were designed on adjacent constitutively expressed exons, in some cases spanning several target exons. If the splicing index was defined for the first exon, two pairs were designed. The first pair was designed between the spliced first exon and the next one. The amplicon will be present in case of normal variant. The second pair was designed between exons after the exon spliced. The amplicon will be present in all cases. PCR reactions were made with the same protocol as previously described. In order to test specificity of primers, we tested them with cDNA from human foetal heart and kidney extracted from RNA (Clontech).

### Statistical analysis

To determine the sample size to the clinical study, we used the result of a pilot study and we performed a chi square sample size with an alpha risk of 0.05% and a desired power of 0.9%. The predicted presence of the spliced product was 90% of haemodialysis patients, 50% of CKD patient and 10% of patient with no CKD. The sample size was calculated to be 29 in each group. Data are expressed as median and [25%–75%] for non-normally distributed values. Statistical analysis was performed with the Prism software (GraphPad Software, Inc., San Diego, CA, USA). Differences were considered significant when p was less than 0.05 and 95% confidence interval. Comparisons between groups were performed with a chi-square test for categorical variables and a one-way analysis of variance for continuous variables. The multivariate analysis was made using logistic regression.

## Results

### Preliminary work: the AS variant of *PKD1* seems associated with CKD

**Figure 1:**
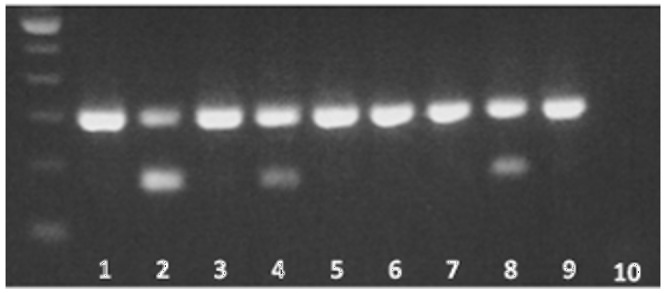
Alternative splicing of exon 30 of *PKD1*. Four patients with polycystic kidney disease but normal kidney function (lane 1, 3, 5, 7) and four relatives with end stage renal disease (Lane 2, 4, 6 and 8) were tested. Lane 9 is positive control, lane 10 negative control of PCR. The non spliced fragment is 300 base pairs and the spliced fragment is 173 bases pairs (bp).

During screening for identification of mutation in patients ADPKD [16], we identified a new AS variant of *PKD1* in PBMCs. This AS variant resulted in the loss of the thirtieth exon and was co-expressed with the wild type form of the transcript. We confirmed the loss of exon 30 by cloning and sequencing (data not shown). The AS was present in 11 of the 12 patients with ADPKD and CKD5D and only in 1/11 healthy controls without CKD (data not shown). The AS could be linked to CKD5 or ADPKD. To distinct between the both possibilities; we compared the occurrence of this AS events in the PBMC of patient with ADPKD with or without impaired eGFR. This AS variant was not found in patients with normal kidney function and was present in 3 over 4 patients with CKD5D (Figure 1). We sequenced exon 30 and intron 29 and 30 to search a polymorphism or a mutation responsible for this AS event. No mutation was found. Thus, the deletion of exon 30 is associated with altered renal function but not with mutation in *PKD1* or polycystic kidney disease status. So, we assumed that this AS variant is largely favoured by impaired kidney function.

### AS variant of *PKD1* is associated with CKD

Thirty eight patients were included in the CKD5D group (HD), 27 in the CKD3-5 group and 31 in the control group. Clinical and biological characteristics were summarized in the table 1. The AS variant was present in 100% of the HD group (38 patients), in 74% of the CKD group (20 patients), and in 35% of the control group (11 patients) (p<0.001). The significant effect of kidney function remained after adjustment for covariates (age, sex, race, ACKD). The odd ratios to present the splicing variant was 44 [IC 95%: 3.5–568] in CKD5D group and 20 [IC 95%: 1.22–334] in CKD3-5 group versus control group. We confirmed that CKD was associated with a specific splicing event of *PKD1* in PBMC.

**Table 1:** Clinical and biological characteristics of the three groups.

		CKD5D (n = 38)	CKD3-5 (n = 27)	Controls (n = 31)	p
**Age (y)**		69 [64–71]	61 [56–64]	66 [59–71]	0.001
**Sex ratio (W/M)**		18/20	17/10	4/27	0.001
**Dialysis Vintage (years)**		7.5 [3]–[18]			
**Kidney diseases**	Glomerulonephritis	11 (32%)	4 (15%)		0.025
	Diabetes	3 (8%)	7 (26%)		
	Vascular Nephropathy	14 (37%)	5 (18%)		
	Interstitial Nephropathy	3 (8%)	8 (30%)		
	Unknown	7 (16%)	3 (11%)		
**Ethnicity (% caucasian)**		34 (89%)	26 (96%)	31 (100%)	ns
**Creatinine (µM)**			253 [128–444]	91 [79–99]	<0.001
**Acquired cystic kidney disease**		26	15	0	ns
**Glomerular Filtration Rate MDRD (ml/min/1.73m^2^)**			20 [9–46]	76 [67–87]	<0.001

For numeric values, data were expressed in median, CI 95%. For clinical values, data were expressed in number (%).

### Exonic microarrays and PCR validation: only one splicing variant highlighted

**Figure 2:**
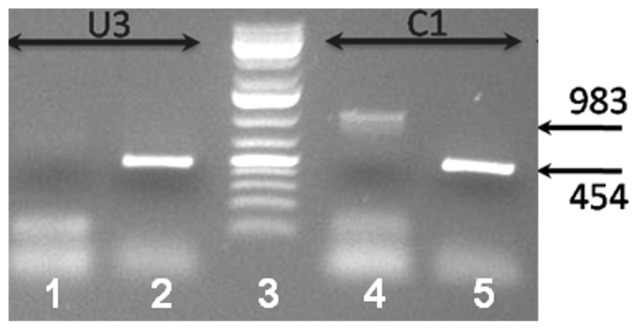
PCR *ADH1B* to explore the aternative splicing of exon 1 in fibroblasts. In normal serum, (C1), the two forms of the transcript is present, lane 4 transcript with exon 1-2-3 (983pb) and lane 5, transcript with exon 2 and 3 (454pb). In uremic serum (U3),the transcript included exon 1 is not present; lane 1 : no transcript, lane 2, transcript with exon 2 and 3.

**Figure 3:**
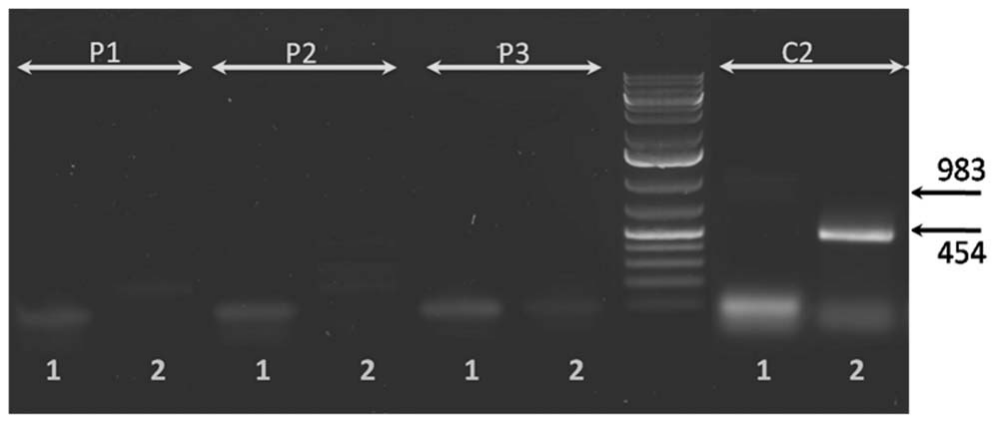
PCR of *ADH1B* to explore the alternative splicing of exon 1 in PBMC. P1, P2, and P3 represent PCR product of three different PBMC from HD patient, C2 represent fibroblast in normal condition. Lane 1 is the PCR for the transcript 1-2-3 and lane 2 is for the transcript 2-3. There is no expression of ADH1B in PBMC (P1, P2, P3) but the gene is expressed in fibroblast (C2).

To identify more alternative splicing events, we performed exonic microarrays analysis of RNA extract from normal fibroblasts incubated with serum from patients with CKD5D compared to fibroblasts incubated with non uremic serum. Uremic serum modified the splicing index of 36 exons probes over 233,164 with absolute splicing index over than 2 and p<0.05. As expected, uremic condition also modified gene expression (data not shown, available in GEO). The splicing index is corrected for the gene expression. The 35 genes corresponding to the 36 probes were summarized in table 2. Only 23 genes were explored for the following reasons: 6 genes were described in NCBI as non-protein coding RNA, the probe designed was the same for 2 genes, one had two exons with significative splicing index, for 4 genes, annotation in database do not allowed us to design primers for RT-PCR, TRA@ is genomic sequence, *CSPG4P5* and *C20orf197* are pseudogenes, *FAM179A* the sequence of the probe is absent from the human genome sequence. So, only 23 genes were explored by RT-PCR: *NCF1C, OVCH1, MYOM3, C10orf92 (2 exons), COL20A1, ROBO2, FBXO10, ACP1, FAM38B, TDRD9, NR2E3, KRT40, FLJ22184, EYS, RASSF2, OPRM1, ADH1B, KIAA1529, SLC16A10, KIAA0319, CNTN4, ADH1A, RTEL1*. Over those 23 genes, only one AS variant was confirmed in *ADH1B* (Figure 2). No absolute splicing index over 2 was found for the probe mapping the exon 30 of *PKD1*. We confirmed the lack of induction, by uremic serum, of the AS of *PKD1* in the fibroblasts by RT-PCR. To confirm the splicing event in patients, we performed an amplification of *ADH1B* on PBMC's mRNA. Unfortunately, *ADH1B* is not expressed (Figure 3) in PBMC whereas *ADH1B* is currently expressed in fibroblast.

**Table 2:** List of genes identified alternatively spliced in fibroblasts by microarrays.

Gene Name (ID)	Splicing Index Uremia vs Normal	p	Splice predicted exon	primers	PCR results
**NCF1C (65417)**	−5.58	0.030	Exon 5	CCACCTCCTCGACTTCTTCA ACACGTCTTGCCCTGACTTT	No differences
**OVCH1 (341350)**	−3.99	0.036	Exon 2	GTTGCTGGTCATCGGCCACCC AGGGGTTGGCCCCGATCCAAA	No differences
**MYOM3 (127294)**	−3.89	0.011	Exon 15	TGACCATCCCCTCACCGCCAA ATGTGGCCCGCACCTCGGAT	No differences
**C10orf92 (54777)**	−2.57	0.006	Exon 29	GAACTCGCCGAGCGTGTCTGG CGGGGCAGGAGTAGGAAGCCCA	No differences
			Exon 34	TTCCTACTCCTGCCCCGAGGA GTGCTGGGCCTGTGCGATCAT	No differences
**COL20A1 (57642)**	−2.50	0.022	Exon 17	GGCGTGCTTGTCTACCAGAT CCTTGAAGAGCGTGAAGGTC	No differences
**ROBO2 (6092)**	−2.42	0.003	Exon1	GCGCAATGCCAGCGACCTTC TGTGGGATATGTGTGGCTGTGTG	No differences
				GGGCATACTCGGGCTGTTTCC AAGCTGGTGGGGCCCATGAA	No differences
**FBXO10 (26267)**	−2.35	0.027	Exon 1	GTGGGCGAGCGCCCTGTG AGGGGCACGTTGTGCAGGAAG CTTGGAGCTGTGGCGCATGAT	No differences
**ACP1 (52)**	−2.23	0.035	Exon 3 (transcrit variant 4)	AAGTCCGTGCTGTTTGTGTG TCAGGGTGGGGTCTAGTCAG CCCATTGCAGAAGCAGTTTT	No differences
**FAM38B (63895)**	−2.22	0.033	Exon 5	TTCCGATACAATGGGCTCTC AACTCGCCTTCAACACCATC	No differences
**TDRD9 (122402)**	−2.18	0.024	Exon 25	GGTTGTATTGGCAGGTGCTT AGGTGCCAGACAGACCAAGT	No differences
**TRA@** **(6955)**	−2.06	0.037	Genomic sequence		
**CSPG4P5 (114817)**	−2.05	0.012	Pseudogene		
**NR2E3 (10002)**	−2.02	0.030	Exon 3	ATGGAGACCAGACCAACAGC AGGTCTCATGGATGCTGTCC	No differences
**KRT40 (125115)**	−2.00	0.028	Exon 4	ACATCCCGATGTCAGACTCC ATTTGCACAGGGTCAGTTCC	No differences
**FLJ22184 (80164)**	−2.00	0.013	Exon 1	CAGGCGACTCTGTGAGGAG CTGAGATCCGGAGTCCCTTC CAAGAGAGACAAGGCGAAGG	No differences
**EYS (346007)**	2.04	0.006	Exon 33	TTGCAGCACCCTCTGTGTGCC GGGCTGCCAACAGGCGTTGT	No differences
**RASSF2 (9770)**	2.09	0.000	Exon 14 (transcrit variant 1)	GAAGGCAAGGCGAAGGGGTGG AGCGGTGGCGTCTGATTCGC	No differences
**OPRM1 (4988)**	2.10	0.013	Exon 6a	CCATTTGGAACCATCCTTTG GAATGGCATGAGACCCTGTT	No differences
**FAM179A (165186)**	2.12	0.036			
**C20orf197 (284756)**	2.13	0.044			
**LOC100192378 (100192378)**	2.17	0.005	non-protein coding RNA		
**NCRNA00202 (387644)**	2.20	0.007	non-protein coding RNA 202		
**C10orf50 (645528)**	2.22	0.013	non-protein coding RNA 264		
**ADH1B (125)**	2.30	0.014	Exon 1	AGACTCACAGTCTGCTGGTGGGC GGTGCGTCCAGTCAGTAGCAGC AGCCTCGCCCCTGGAGAAAGT	SPLICE EXON HIGHLIGT
**KIAA1529 (57653)**	2.31	0.001	Exon 2	CCGACACCTTGAGCGCCGTT CGCACTTCTCGGGCAGCGAT	No differences
**SLC16A10 (117247)**	2.52	0.048	Exon 5	CCTGCGGCTGCTCCTTTGCAT TAAGGGGAGGGACTCCAGCGAG	No differences
**LOC254559 (254559)**	2.53	0.020	non-protein coding RNA		
**KIAA0319 (9856)**	2.58	0.048	Exon 10	AAGCCACCCCACAGACTACCAA TCCCAGGACCCAGGGACCACT	No differences
**CNTN4 (152330)**	2.74	0.013	Exon 22	TGGTGGCGGAGGCAGCAAATC TGCCATCTCCTCCGTCGCTG	No expression
**ADH1C (126)**	2.75	0.010	Identic probe than ADH1A		
**LOC440910 (440910)**	2.77	0.008	non-protein coding RNA		
**ADH1A (124)**	2.83	0.009	Exon 1	CTCCTGGTCTGCAGAGAAGACAGA GTCCCCTGAGGATTGCTTACATCG ATGAGGCAGCCGGCATCGTG	No differences
**LOC440905 (440905)**	3.10	0.007	non-protein coding RNA		
**TNFRSF6B (8771)**	4.43	0.001	Identic probe than RTEL1		
**RTEL1 (51750)**	4.44	0.001	Exon 14	CTGAAGGGGACAGTCGTGAT GAGCCGTCCTCCGGTGACCA	No differences

## Discussion

Although AS is the major source of protein diversity in human being, it has not been extensively documented with respect to pathology, excepted for cancer [19]. We confirmed the existence of AS events, in vivo and in vitro correlated with CKD, but this level of control of gene expression is infrequent. Exon microarrays are designed to study the exon-level gene expression of each exon over the expression of all the others of the same gene. Probes are designed to target all exons within each gene. The expanded density of the 2×400K format allows the inclusion of probes to detect exons as short as 35 nucleotides in length and and the inclusion of probes corresponding to the 5′ and 3′ untranslated regions [20]. Over 233 164 probes, uremic serum modified splicing index of 36 exons. Over these 36 exons, only one splicing variant was found associated with uremic condition. Our confirmation rate of AS variant in this study is lower than previously observed (1 over 23). The statistical methods (with no correction) can explain this lack of specificity. Few studies were published using the same exon microarrays [21]. Zhang et al, did not report the rate of confirmation [21]. However they highlighted only 3 splicing variants over 193 predicted by the array. Since Johnson and co-worker [22] published the first human genome-wide microarray that utilized exon-exon junction probes, others exon microarray platforms have emerged. Confirmation rate of AS variant seems to vary from 30% to 73% [23], [24]. One limit of our microarrays analysis is that we used only one cell type to study splicing events, in vitro. Our results must be confirmed in other cell types. Fibroblasts were chosen because of their ubiquity the exposure to uremic toxins in vivo, the lack of organ specificity in order to study a common cellular mechanism. Moreover; fibroblasts are primary cells but can be cultured until ten passages. So we could use the same cells for all experiments. In addition, primary PBMC incubated in uremic serum showed increased mortality as described by others [18]. Furthermore, cultured fibroblasts are often used to explore splicing events in cancer [25].

In fibroblast in uremic condition, we did not identify the *PKD1* exon 30 splicing event in microarray and confirmed in RT-PCR. This could be relied to various issues. First this AS variant is restricted to the PBMC under uremic conditions, and second the splicing event does not occur in vitro but need another signal than uremic soluble environment, like interactions with modified matrix.

Deletion of exon 30 could lead to the production of a truncated Polycystin-1 (PC-1) after the second transmembrane domain in case of transcription. The function of PC-1 in PBMC is not critical. So, clinical and functional significance of this AS variant is difficult to approach. Nevertheless, the AS of *PKD1* could be of interest if it is expressed in the kidney. It could provide a way to explain the high incidence of acquired cystic kidney disease observed in patients with CKD [26]. Unfortunately, our clinical study did not put in light a statistical association between ACKD and the AS of *PKD1*.

The protein encoded by the gene found alternatively spliced in vitro, *ADH1B*, is a member of the alcohol dehydrogenase family. Members of this enzyme family metabolize a wide variety of substrates, including ethanol, retinol, other aliphatic alcohols, hydroxysteroids, and lipid peroxidation products. This encoded protein, consisting of several homo- and heterodimers of alpha, beta, and gamma subunits, exhibits high activity for ethanol oxidation and plays a major role in ethanol catabolism. The putative protein encoded by the splicing variant lacks sequences encoded by the first two exons so the 40 first amino-acids. It represents the CRA-b isoform (GenBank: EAX06097.1). This isoform is not related to any pathology or disease. Unfortunately, we were not able to confirm the splicing event in vivo. *ADH1B* is not expressed in the PBMC. We confirmed previous results from the literature [27]. Its expression seems to follow the same pattern in hepatocytes and fibroblasts even if the expression in the later is weaker [28]. Polymorphisms in this gene are associated to the risk of premature coronary artery disease [29] or alcoholism[30]. So, it is difficult to understand the clinical relevance of this splicing event as it was for the AS variant of *PKD1*.

In conclusion, regulation of the AS machinery does not seem to be a major mechanism to modulate gene expression in fibroblast incubated in uremic condition. Despite, the low frequency of the event, we identified two spliced variants in two different cell types. So, the possibility of alternative splicing as a mechanism leading to modification of gene expression during CKD should be address systematically.
